# Cancer

**Published:** 1901-08-10

**Authors:** 


### Cancer.

After tuberculosis we come to cancer. Those who watch the actual growing points of knowledge are aware that tuberculosis has already become a matter of administration, to be discussed by town councillors and public officials, and that the centre of scientific interest has moved forwards and is now focused upon that group of diseases which we commonly speak of as cancer. It is probable that the King, with his usual power of getting at the heart of things, was fully aware of this when, in addressing the foreign delegates to the Tuberculosis Congress, he said: "There is still one other terrible disease which has up to now baffled the scientific and medical men of the world, and that is cancer. God grant that before long you may be able to find a cure for it, or check its course." And that is what men are now working for.

At every stage of the world's history man's powers have been limited by his tools; in all the arts what a man effects is largely determined by the appliances to which he has access, and it is not to be wondered at that with the new bacteriology to hand as a means of investigation, and with the new surgery available as a means of treatment, the men of to-day should have applied these "tools" to the great problem of cancer. Thus it is that investigators are just now chiefly engaged in hammering out the question of infection, and that extirpation is the keynote of all that concerns the treatment of the disease. At the present moment these are the lines along which progress is being chiefly made; but that is because it is along these lines that the new means now at our disposal make it most easy to advance. We must not, then, speak with positiveness, or assert that this will be the direction which the great discovery will take—when at last the man shall arise to point the way.

That cancer is infective within the body of the patient who is attacked by the disease is obvious enough; in fact, it is from its well-recognised tendency to eat its way into surrounding tissues and to infect distant organs that it has gained its name as a "malignant" disease, and for a long time men have suspected that cancer might be infectious also from man to man. During recent years a considerable number of observations have been collected which go to show not only that cancer is far more prevalent in some districts than in others, but that the disease so persistently haunts certain houses as to give "infectionists" a strong basis for their faith. It is to be noted, however, that all these well-authenticated cases of "cancer houses" or "cancer localities," and all the instances in which successive cases of cancer have occurred among people living together, would be equally well explained on the hypothesis which has been put forward by Mr. D'Arcy Power to the effect that the germ of the disease is not a thing transferable directly from man to man, but is a *contagium vivum* which lives in some intermediate host, be it animal or vegetable, from which under certain favouring conditions it finds its way to a spot suitable for its growth in the body of its human victim.

What is this parasite, if such a parasite exists, has long been a matter of anxious search. Again and again have investigators thought that they had laid their fingers on it, only, on further investigation, to be disappointed. But certain facts have been discovered which can hardly be gainsaid. Mr. Plimmer, of St. Mary's Hospital, to whom we owe much in regard to this line of investigation, has shown that there are certain intracellular bodies which for the time may be called, as Metchnikoff calls them, "parasitic protozoa," which are found constantly at the periphery, the growing part of the tumour in cancer, and are constantly absent in other diseased conditions of the cell. He has shown also that these bodies can be cultivated outside the body, and that these cultures when introduced into certain animals can cause death with the production of tumours, and that cultures from these tumours will produce again similar tumours. Thus he has gone a long way. But these tumours were different from those from which the "parasites" were originally taken; and here for the present the argument for infection stands—broken off just short of proof.

As for treatment the pointing hand of science is much more certain. Whether the stimulus to that epithelial overgrowth and invasion which is the characteristic feature of cancer be a parasite or some other irritation, or indeed be merely a defect of the tissues by which epithelial intrusion ought to be prevented, the fact remains that it is a local affair at first. What modern surgery teaches is that, if the local mischief can but be removed early enough and thoroughly enough, the patient may live on without recurrence to the term of his natural life; and thus, pending the time when the pathologists shall throw more light upon the nature of cancer, our sole and whole duty as practitioners in regard to it is to discover the disease as early as we can and then remove it without delay.

